# The Effects of Medical Male Circumcision on Female Partners’ Sexual and Reproductive Health

**DOI:** 10.1007/s11904-022-00638-6

**Published:** 2022-11-11

**Authors:** Supriya D. Mehta

**Affiliations:** 1 Division of Infectious Disease Medicine, Rush University College of Medicine, Chicago, IL USA; 2 Division of Epidemiology & Biostatistics, University of Illinois Chicago School of Public Health, Chicago, IL 60612 USA

**Keywords:** Male circumcision, Female sex partners, Sexual and reproductive health, Penile microbiome, Vaginal microbiome

## Abstract

**Purpose of Review:**

Voluntary medical male circumcision (VMMC) reduces the risk of HIV acquisition by 60% among heterosexual men, provides protection against certain sexually transmitted infections (STI), and leads to penile microbiome composition changes associated with reduced risk of HIV infection. Intuitively, the benefits of VMMC for female sex partners in relation to STI are likely and have been evaluated. The purpose of this review is to examine emerging findings of broader sexual and reproductive health (SRH) benefits of VMMC for female sex partners.

**Recent Findings:**

Systematic reviews find strong evidence for beneficial effects of VMMC on female sex partners risk of HPV, cervical dysplasia, cervical cancer, and with likely protection against trichomoniasis and certain genital ulcerative infections. Few studies assess the direct impact of VMMC on the vaginal microbiome (VMB), though several studies demonstrate reductions in BV, which is mediated by the VMB. Studies are lacking regarding male circumcision status and outcomes associated with non-optimal VMB, such as female infertility and adverse pregnancy outcomes. VMMC has positive effects on women’s perceptions of sexual function and satisfaction, and perceptions of disease risk and hygiene, without evidence of risk compensation.

**Summary:**

VMMC has consistent association with a broad range of women’s SRH outcomes, highlighting the biological and non-biological interdependencies within sexual relationships, and need for couples-level approaches to optimize SRH for men and women. The paucity of information on VMMC and influence on VMB is a barrier to optimizing VMB-associated SRH outcomes in female partners.

## Introduction

It is well-known that voluntary medical male circumcision (VMMC) reduces the risk of heterosexually acquired HIV in men by 60% [1–3] and provides protection against some sexually transmitted infections (STIs) in men (genital ulcerative infections, HPV) [4, 5]. The ways in which VMMC protects against these infections have been described [4], but briefly, plausible biological mechanisms include reduced epithelial disruption of the preputial mucosa, increased keratinization reducing mucosal access of pathogens, and reduced environmental survival of pathogens through removal of the sub-preputial space. Additionally, VMMC causes a major shift in the penile microbiome composition, with substantial decreases in the prevalence and abundance of anaerobic bacteria that are often associated with Bacterial vaginosis (BV) in female sex partners [6] and are associated with genital mucosal inflammation in men [7, 8].

Given the intrinsic transmissibility of STIs and exchangeability of the genital microbiota during sex, it is intuitive that benefits of VMMC would directly transfer to female sexual partners. In addition to these proximal biomedical benefits, it is possible that VMMC could influence more distal or non-biological outcomes in female sex partners. The purpose of this review is to examine the association of VMMC with more broadly considered measures of sexual and reproductive health of female sex partners, and to highlight gaps and areas for future research, focusing on the literature published 2017–2022). We begin with summary of what is known and emergent additions to the literature, and then consider additional outcomes related to vaginal microbiome and sexual functioning, satisfaction, and quality of life.

## Impacts of Medical Male Circumcision on Women’s Sexual and Reproductive Health: What is Known

A systematic review of the impact of medical male circumcision on biomedical outcomes in female partners by Grund et al. [9••] including publications through April 2016 found high-quality evidence for the protection of male circumcision against cervical dysplasia, cervical cancer, HSV-2, *Chlamydia trachomatis*, and syphilis in female sex partners. Subsequently published systematic review by Morris et al. [10], which included publications through August 2018, echoes these findings for HSV-2, syphilis, and HPV; this can be expected as many articles included in these two reviews overlap.

Grund et al. determined the consistency and direction of evidence were indeterminate or low for impact of VMMC on female partner Bacterial vaginosis (BV), candidiasis, dysuria, genital warts, gonorrhea, HIV, HPV, *Mycoplasma genitalium,* non-specific genital ulcers, trichomoniasis, and vaginal discharge, noting these evaluations were limited by small number of studies, variable quality, and limited generalizability [9••]. In contrast, the review by Morris et al. was more confident in the association between VMMC and reductions in women’s risk of oncogenic HPV, *Trichomonas vaginalis*, and BV [10]. Reduction in genital ulcer disease was assessed as potential, while findings for impact of male circumcision on women’s risk of HSV-2, *C. trachomatis*, syphilis, HIV, and candidiasis were classified as mixed. Similar to Grund et al., Morris et al. reported no association between male circumcision status and female partner risk of *N. gonorrhoeae*, *M. genitalium*, dysuria or vaginal discharge.

Published subsequent to these two systematic reviews, a household survey evaluation of VMCC on laboratory-detected STIs in 4,640 women in South Africa observed protective effect against HSV-2 (adjusted odds ratio [aOR] = 0.71; 95% CI: 0.53–0.95) and HIV (aOR = 0.66; 95% CI: 0.49–0.90) for female partners, adjusted for women’s age, partner’s age, educational attainment, income, relationship status, condom use, and alcohol consumption prior to sex [11••]. However, this household survey observed no association between male circumcision status and *N. gonorrhoeae, C. trachomatis, T. vaginalis,* or *M. genitalium* [11••]. Also published subsequent to the reviews by Grund et al. and Morris et al., authors of the VOICE (Vaginal and Oral Interventions to Control the Epidemic) trial evaluated male partner circumcision status and incident STI and sexual practices in female partners [12••]. VOICE was a multicenter (15 sites across South Africa, Uganda, and Zimbabwe) randomized, placebo-controlled trial of oral and topical PrEP to prevent HIV acquisition among 5,029 HIV uninfected women aged 18–45. In this analysis, investigators observed reduced risk of syphilis (adjusted hazard ratio = 0.52; 95% CI: 0.27–1.02) among women with circumcised sex partners, adjusted for age, educational attainment, and marital status. The VOICE trial did not find association of male circumcision status with *N. gonorrhoeae* or *C. trachomatis* incidence. There was no evidence of sexual risk compensation among women with circumcised partners, as measured by frequency of condomless sex or number of sex acts. These large studies with objective markers of STIs and comprehensive measures of behavior strengthen the evidence for VMMC’s protective effect against STIs in female sex partners.

The protective effect of VMMC on cervical cancer and HPV in female partners has been evaluated in great depth in systematic reviews [9••, 13]. Within heterosexual couples, genotype specific HPV load is correlated between partners [14], explaining the conferred benefits of VMMC on HPV acquisition in men to female sex partners. Based on the strong impacts of VMMC on HPV acquisition in men (53% reduction) and HPV clearance in men (56% increase), results of modeling demonstrate that even in the absence of expanded HPV vaccination, by the year 2067, VMMC scale up in Uganda could decrease cervical cancer incidence from 31.2 cases per 100,000 women to 25.3 cases per 100,000 women [15]. In the setting of 45% vaccination coverage, or HPV screen-and-treat without vaccination, VMMC would still lead to significant declines in cervical cancer incidence and deaths averted. A modeling study in Tanzania estimates that between 1995 and 2020, VMMC prevented 2,843 cervical cancer cases and 1,039 cervical cancer deaths, and by 2070 could reduce incidence from 55.1 cases to 39.9 cases per 100,000 women [16]. Thus, the strong protective effect of VMMC against HPV acquisition and persistence in men has potential to improve distal outcomes of reduced cervical cancer incidence and mortality in female partners.

In summary, there is consistent evidence for protective effect of VMMC against certain STIs among female sex partners. It is unlikely that randomized controlled trials will be undertaken again; to build evidence and understanding, prospective studies with etiologic measures of STI incidence in women should incorporate objective measure of male partner circumcision status as an efficient way of generating this needed data. Ideally, these same studies would include measure of STIs in male partners to determine direct effects, though STI incidence studies in men are often of low priority, due to most adverse reproductive and pregnancy sequelae manifesting in women. Specific STIs that require additional study include *M. genitalium* and *T. vaginalis*. *M. genitalium*, largely excluded from public health surveillance systems, yet associated with preterm birth [17], having high rates of cervicitis and pelvic inflammatory disease [18], reliance on molecular approaches for diagnosis [19], and growing antimicrobial resistance [20] is an STI of importance. A technical consultation to the NIH determined clinical trials for screening and treatment of *M. genitalium* infections in women and their sex partners to be a priority for improved reproductive health [21]. Like *M. genitalium¸T. vaginalis* is largely excluded from public health surveillance and is subject to antimicrobial resistance [20]. There is growing evidence for the disproportionate impact of *T. vaginalis* on women of African descent, and contribution to broad reproductive health disparities [22]. Generating understanding of the effect of male circumcision status on these two STIs addresses a knowledge gap and is aligned with priority areas for STI prevention and treatment.

## Impacts of Medical Male Circumcision on Women’s Sexual and Reproductive Health: Emerging Evidence

### Effect of VMMC on Female Partner’s Vaginal Microbiome

Male circumcision status modifies the penile microbiome, which may in turn influence the vaginal microbiome. Numerous studies demonstrate that anaerobic bacteria are recovered in greater frequency and abundance from uncircumcised men [23••]. With the loss of the sub-preputial environment, VMMC has been shown to reduce the relative abundance, prevalence, and load of anaerobic bacteria [24–26], such as *Peptoniphilus, Prevotella, Finegoldia, Porphyromonas, Dialister, Mobiluncus,* and *Gardnerella*, many of which are enriched among women with BV [27]. This change in penile microbiome composition following VMMC is likely transferred to female sex partners.

**Fig. 1:**
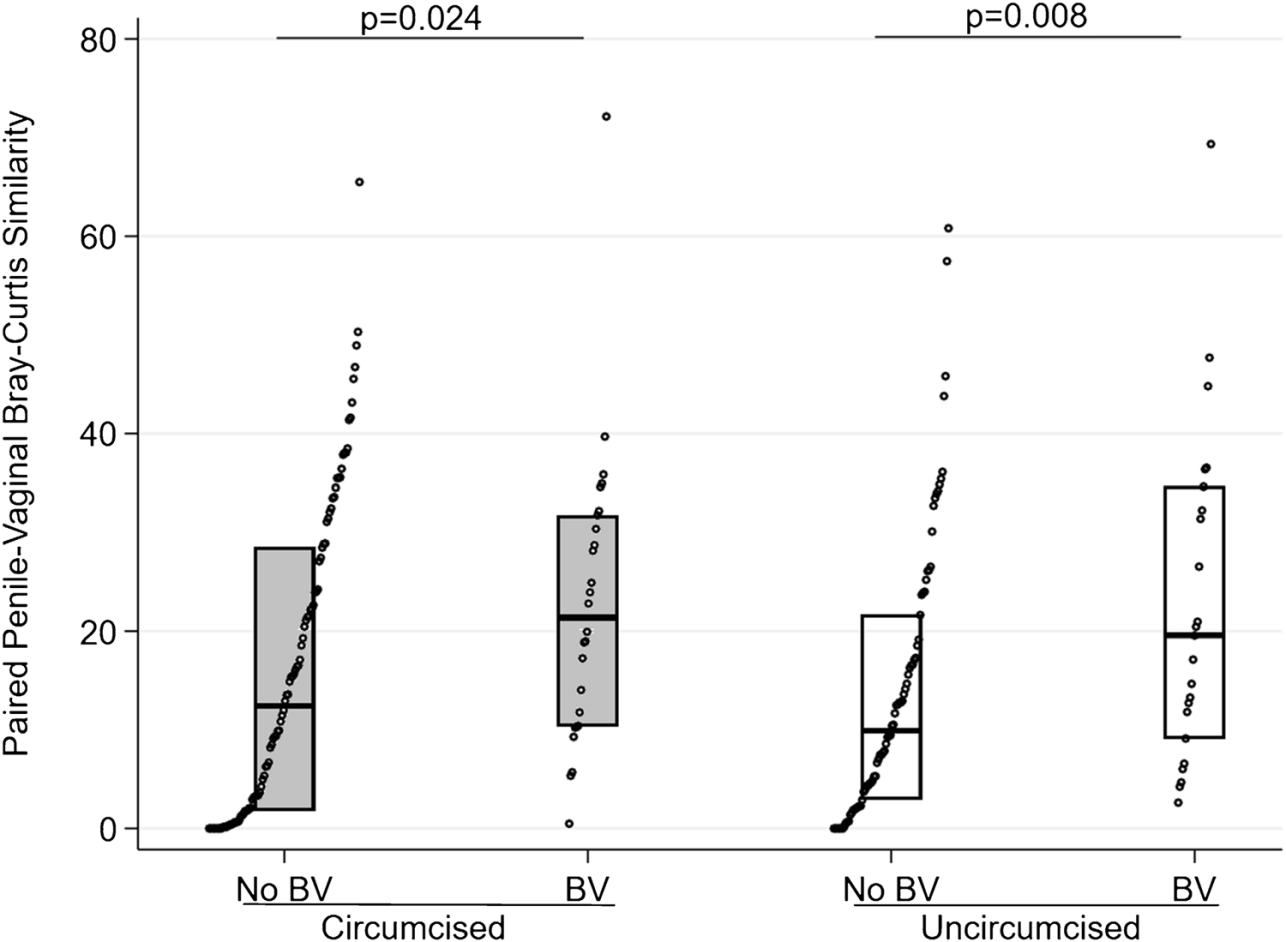
Within couple paired Bray–Curtis similarity of penile and vaginal microbiome communities at baseline among a community recruited cohort of Kenyan couples. Legend: The y-axis represents the paired Bray–Curtis similarity of within-couple penile and vaginal microbiome communities. The x-axis represents subgroups in which the male partner is circumcised (light gray) or uncircumcised (white), and in which the female partner has BV (Nugent score 7–10) or no BV (Nugent score 0–6). The Wilcoxon rank sum p-value comparing similarity between groups is indicated at the top of the figure

In our study of 168 community-recruited couples in Kisumu, Kenya, in which the female partner did not have BV at baseline, the cumulative incidence of BV over one year was 27% for women with a circumcised partner, as compared to 37% among women with an uncircumcised partner (relative risk = 0.69) [6]. We demonstrated that penile microbiome composition at baseline highly accurately predicted incident BV (80.7% sensitivity, 74.6% specificity, 77.5% accuracy), even 6 to 12 months after penile microbiome assessment. Unsurprisingly, penile taxa predictive of incident BV were parallel to vaginal taxa associated with BV: *Gardnerella vaginalis, Sneathia sanguinegens, Dialister, Lactobacillus crispatus, Lactobacillus iners,* and *Parvimonas*. Circumcision status was not a highly influential variable in predicting BV, once assessed in conjunction with penile microbiome. Other studies have observed concordance between vaginal and penile or seminal microbiome compositions [28, 29], though unlike our study, these did not assess directionality, whereby penile microbiome composition precedes BV or vaginal microbiome change. The study by Mandar et al. did not assess the impact of circumcision status [29], but that of Zozaya et al. [28] observed that penile and vaginal bacterial communities were more similar in couples where the woman had BV than in couples where the woman did not have BV, and this did not differ by men’s circumcision status. Using baseline data from our cohort in Kenya [6], we also observed within-couple paired Bray–Curtis similarity was significantly increased in couples where the woman had BV, and this did not differ by men’s circumcision status (Fig. 1). While beyond the scope of the current paper, analysis is needed regarding factors influencing variation over time in penile and vaginal microbiome sharedness, how these factors may differ by male circumcision status, and whether this precedes or is coincident with BV.

We are unaware of other studies directly evaluating the influence of men’s circumcision status on female partner’s vaginal microbiome composition, but studies have examined the effect of circumcision status on BV in female partners. The systematic review by Grund et al. identified 8 such studies. Excluding two studies that included especially high-risk women, there was high consistency evidence for a protective association between medical male circumcision and BV [9••]. A subsequently published review by Morris et al. also summarizes that most evidence indicates a reduced risk of BV for women with circumcised male partners [10]. Unfortunately, the publications subsequent to reviews by Grund et al. and Morris et al. did not assess BV in relation to male circumcision status [11••, 12••]. The impact of VMMC on female partner BV status is intuitive, given that BV represents a shift in the vaginal microbiome from one that is predominantly *Lactobacillus* dominant (in particular *L. crispatus*) to one that is diverse and replete with anaerobic bacteria, as listed above*.* However, quantification of how the penile microbiome mediates the vaginal microbiome and how this mediation varies by circumcision status is lacking. This is relevant as trials are completed [30] or underway to assess the effect of male partner treatment with antibiotics to reduce BV recurrence. If antibiotic effect on the penile microbiome (e.g., specific taxa altered, magnitude, and duration of change) varies by men’s circumcision status, this is critical to understanding overall effects and choice of treatment regimen.

The genital tract microbiome has been shown to have a strong role in women’s fertility and pregnancy outcomes. In a systematic review by Vitale et al., *L. crispatus* was associated with better fertility, while *C. trachomatis, Gardnerella vaginalis, Ureaplamsa* spp., and asymptomatic BV were negatively associated with fertility [31]. Systematic review also demonstrates that BV and a diverse, non-*Lactobacillus* dominated vaginal microbiome are associated with increased likelihood of adverse pregnancy outcomes (pre-term birth, premature rupture of membranes) [32]. Despite the correlation between male circumcision status and female partner vaginal microbiome, we have not identified studies that assessed male circumcision status in relation to women’s fertility and pregnancy outcomes. Understanding the relationship between circumcision status, penile microbiome composition, and fertility and pregnancy outcomes in female partners may lead to identification of therapeutic avenues that optimize these outcomes.

### Male Circumcision Status and Female Partner Sexual Satisfaction

In randomized controlled trials, VMMC is associated with improvement in measures of men’s sexual satisfaction (e.g., penile sensitivity, ease of reaching orgasm) [33, 34], and no declines in sexual function. A meta-analysis examining medically indicated and non-medically indicated circumcision and association with sexual function and satisfaction affirms these findings: in high quality publications, male circumcision was not associated with inferior sexual function or satisfaction for men [35]. While an abundance of literature signals clearly that there are likely benefits and no declines in male sexual function and satisfaction, there is less data available on the effects of male circumcision on female sex partner sexual satisfaction and function.

A systematic review by Grund et al. summarizes the evidence on the association between male circumcision status and women’s sexual satisfaction and sexual function [36]. From 7 studies published through 2017, there was high consistency of evidence for a positive association between male circumcision and women’s sexual satisfaction, though measures were varied across publications. The positive association varied geographically, with female respondents in Canada and Denmark reporting higher satisfaction with uncircumcised partners, though sampling and recruitment biases were present. This systematic review observed only one study from Denmark examining the association between male circumcision and women’s sexual function, and classified this as indeterminate evidence. However, other studies measured individual components of sexual function (e.g., orgasm ease, arousal, pain, lubrication), and found associations in varying directions, but were of low or medium quality. In the only study reviewed that had a high-quality score, among female sex partners of men participating in the randomized controlled trial of VMMC in Rakai, Uganda, the majority of women reported no change (57.3%) or improvement (39.8%) in sexual satisfaction, with just 2.9% reporting less sexual satisfaction, after their partners were circumcised [37].

In our prospective cohort study of 252 heterosexual couples in Kenya, we measured sexual quality of life using standardized tools in men and their female partners [38]. While sexual quality of life was nearly 10% lower than the mean for women with BV and recent sex, men’s circumcision status was not directly associated with female partners’ sexual quality of life. In this survey, we also asked women how men’s circumcision status could affect their perceived risk of HIV, STIs, enjoyment of sex, vaginal cleanliness, and injuries to the vagina (Table 1). The perception of the majority of women, even if their male partner was uncircumcised (as determined by clinician exam), was that there were sexual benefits to having a circumcised male partner: less risk of HIV, STI, and vaginal injuries, and more enjoyment of sex and easier to keep the vagina clean. Despite this perception of reduced risk of HIV and STIs with circumcised male sex partners, we did not observe increased frequency of not using condoms, multiple sex partners, or number of sex acts among women with circumcised male partners (Table 1).

**Table 1:** Distribution of women’s reported perceptions and sexual practices by clinician-determined male sex partner circumcision status

Women’s reported responses^1^	Male partner is circumcised *N* = 112 *n* (%)	Male partner is uncircumcised *N* = 140 *n* (%)	Chi-square *p*-value^2^
Perceptions related to sexual practices and men’s circumcision status			
It is easier for women to get HIV infected if the man is…?			
Circumcised Uncircumcised No difference/Don’t know	5 (4.5) 103 (92.0) 4 (3.6)	5 (3.7) 122 (89.7) 9 (6.6)	0.569
Women enjoy sex more if the man is…?			
Circumcised Uncircumcised No difference/Don’t know	92 (90.2) 7 (6.9) 3 (2.9)	128 (97.0) 1 (0.8) 3 (2.3)	0.035
It is easier for women to keep their vagina clean if the man is…?			
Circumcised Uncircumcised No difference/Don’t know	102 (92.0) 5 (4.5) 4 (3.6)	125 (91.2) 6 (4.4) 6 (4.4)	0.953
It is easier for women to get vaginal injuries if the man is…?			
Circumcised Uncircumcised No difference/Don’t know	10 (9.4) 95 (88.8) 2 (1.9)	13 (9.6) 116 (85.9) 6 (4.4)	0.586
It is easier for women to get sexually transmitted disease if the man is…?			
Circumcised Uncircumcised No difference/Don’t know	3 (2.7) 105 (93.8) 4 (3.6)	3 (2.2) 128 (93.4) 6 (4.4)	1.00
Women’s reported sexual practices			
A condom was used at last sexual intercourse	16 (14.3)	27 (19.3)	0.295
Frequency of condom use, past 6 months			
Never Sometimes Always	72 (64.3) 36 (32.1) 4 (3.6)	87 (62.1) 37 (26.4) 16 (11.4)	0.060
Two or more sex partners, the past 12 months	9 (8.0)	8 (5.7)	0.465
Median number of sex acts past 7 days (IQR)	2.0 (1.0–3.0)	2.0 (1.0–3.0)	0.577

^1^Not all cells sum to N due to missing responses; ^2^Fisher’s exact test applied where cell size *n* < 5. Wilcoxon rank sum test applied for comparison of number of sex acts. *IQR* interquartile range

## Conclusions

Given the centrality of vaginal microbiome health to numerous sexual and reproductive health outcomes for women, pregnancy outcomes, and neonates, the paucity of information on male circumcision status and influence on vaginal microbiome is a critical barrier to optimizing these health outcomes. There is need to characterize the penile microbiome, determining what is optimal and non-optimal in terms of composition and function, similar to what has been achieved for the vaginal microbiome [39••]. Understanding how the penile microbiome influences the vaginal microbiome and subsequent health outcomes is necessary for identifying, developing, and prioritizing therapeutic options (such as antibiotic treatments or live biotherapeutics), and whether these need to be modified according to men’s circumcision status, to improve outcomes in men and their female partners.

The consistently demonstrated role of men’s circumcision status in women’s sexual and reproductive health outcomes highlights the biological and non-biological interdependencies of reproductive health within sexual relationships. Translation of these concepts to a *couples-level approach* may lead to increased gains and better optimization of reproductive health and outcomes. For example, couples-based approaches to HIV prevention are effective at increasing HIV testing uptake and ART uptake [40], and couples-based STI testing/partner delivered treatment are effective at reducing re-infection [41]. Involving men in antenatal care has also been shown to improve women’s engagement in antenatal care [42]. These examples highlight that couples-based approaches can be leveraged to achieve greater improvement in women’s sexual and reproductive outcomes. Generating evidence that characterizes and creates broader understanding of men’s circumcision status on women’s sexual and reproductive health outcomes may lead to expanded and improved interventions.
